# Upper Critical Field Based on a Width of ΔH = ΔB region in a Superconductor

**DOI:** 10.1038/s41598-020-61905-3

**Published:** 2020-03-25

**Authors:** H. B. Lee, G. C. Kim, Byeong-Joo Kim, Y. C. Kim

**Affiliations:** Department of Physics, Pusan National University, Busan, 46241 Korea

**Keywords:** Materials science, Physics

## Abstract

We studied a method of measuring upper critical field (H_*c*2_) of a superconductor based on a width of ΔH = ΔB region, which appears in a superconductor that volume defects are many and dominant. Here we show basic concepts and details of the method. Although H_*c*2_ of a superconductor is fixed according to a kind of superconductor, it is difficult to measure H_*c*2_ experimentally. Thus, results are different depending on experimental conditions. H_*c*2_ was otained by a theory on a width of ΔH = ΔB region, which is that pinned fluxes at volume defects are picked out and move into an inside of the superconductor when the distance between pinned fluxes is the same as that at H_*c*2_ of the superconductor. H_*c*2_ of MgB_2_ obtained by the method was 65.4 Tesla at 0 K, which is quite same as that of Ginzburg-Landau theory. The reason that H_*c*2_ obtained by the method is closer to ultimate H_*c*2_ is based on that F_*pinning*_/F_*pickout*_ is more than 4 when pinned fluxes at volume defects of 163 nm radius are depinned, which means that the H_*c*2_ is less sensitive to fluctuation. The method will help to find the ultimate H_*c*2_ of volume defect-dominating superconductors.

## Introduction

Substantially, upper critical field (H_*c*2_) is difficult to measure at 0 K in a type II superconductor. It is because H_*c*2_ of a type II superconductor is very high and ambiguous besides difficulty of temperature control. When a high magnetic field is applied, most of the fields penetrate into the inside of the superconductor, which result in extremely low diamagnetic property by 4*π* M = B  −  H, where M, B and H are magnetization, magnetic induction and applied magnetic field, respectively.

Nevertheless, knowing the ultimate H_*c*2_ of a superconductor is important, which is defined as H_*c*2_ in the ideal state. Practically, it is important to know how high the superconductor maintains its superconductivity in magnetic field, and more important thing is that H_*c*2_ of a superconductor tell us one of important parameters of the superconductor, which is coherence length (*ξ*) (Φ_*o*_/2*πξ*^2^ = H_*c*2_, Φ_*o*_ is flux quantum which is 2.07 × 10^−7^ G⋅cm^2^)^1^.

There are roughly three methods to obtain H_*c*2_ of a superconductor. The first is the method of flowing currents after applying a high magnetic field and checking voltages (supercurrents method). Many researchers are using this method, and the results are relatively reliable^2,3^. However, there is much controversy for which point between the onset and the offset should be regarded as H_*c*2_, and result voltages are also influenced by current density^4^. In addition, in order to make results reliable, measured value should be at least obtained at 5 K. A great deal of efforts to set the equipment are required because H_*c*2_ at 5 K is usually not small. Nonetheless, Gurevich *et al*. used this method measuring H_*c*2_ of MgB_2_ film and reported that H${}_{c2}^{\parallel }$ and H${}_{c2}^{\perp }$ is approximately 48 Tesla (T) and 34 T at 0 K, respectively^5^.

The second method is to measure critical temperature (T_*c*_) and determine H_*c*2_ by theory (H_*c*2_ = 1.83T_*c*_)^6^. Theoretically, H_*c*2_ of MgB_2_ is approximately 68.6 T at 0 K when critical temperature (T_*c*_) is 37.5 K. The third is to extrapolate H_*c*2_ to 0 K after H_*c*2_ was checked at various temperatures in field dependence of magnetization curve (M-H curve). The extrapolation uses the property that H_*c*2_ of a superconductor increases when the temperature decrease (M-H curve method). Of course, this method is convenient, but there is a problem that a limit of the magnetic field exists in equipment and the result are hard to be believed if applied magnetic field does not increases carefully in high magnetic field region. Therefore, the reliability is low.

**Figure 1:**
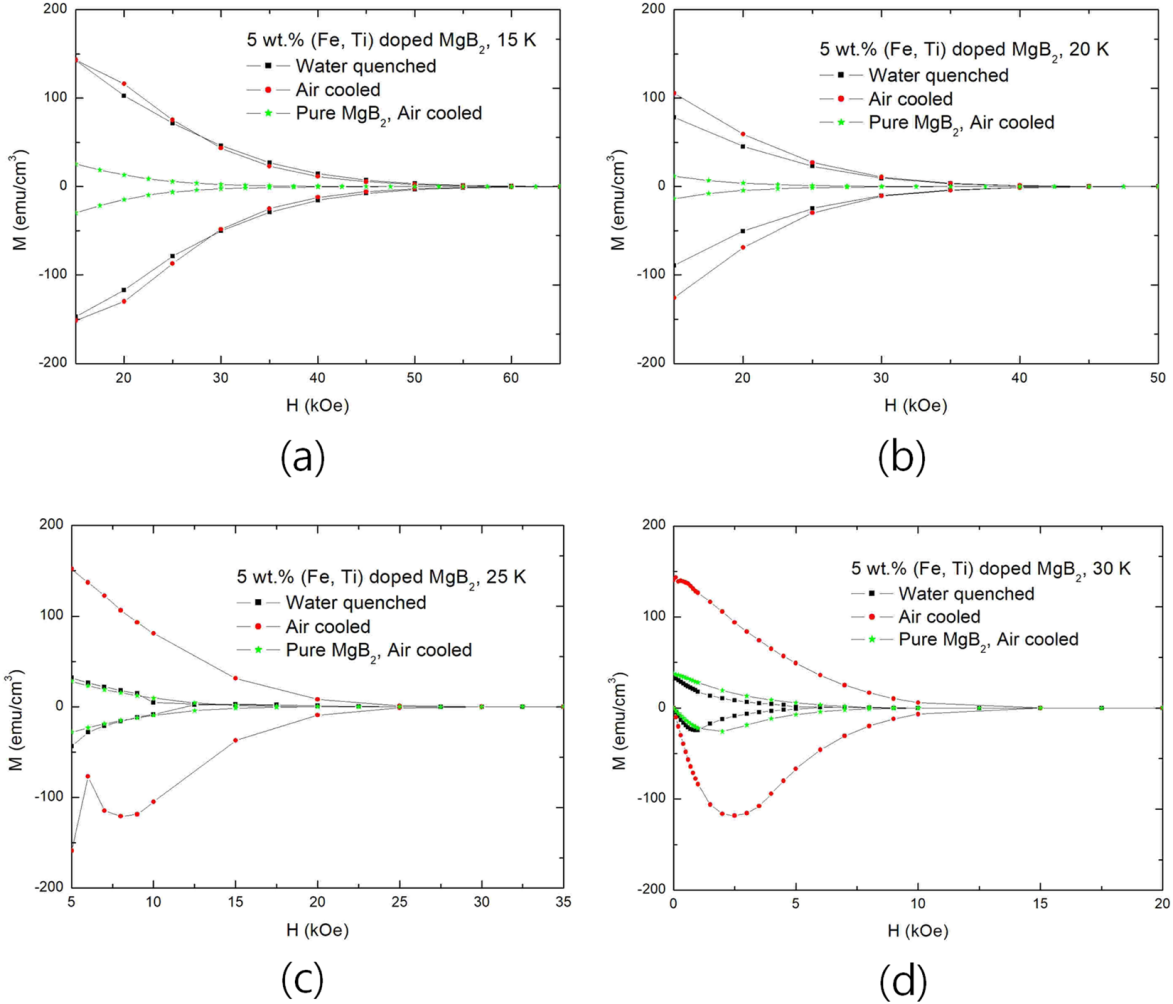
Field dependences of magnetization (M-H curves) of pure MgB_2_ and 5 wt.% (Fe, Ti) doped MgB_2_ at various temperatures. M-H curves are cut to emphasize H_*c*2_ of specimens. Pure MgB_2_ was air-cooled. And 5 wt.% (Fe, Ti) doped MgB_2_ were air-cooled and water-quenched, respectively. (**a**) M-H curves at 15 K. (**b**) M-H curves at 20 K. (**c**) M-H curves at 25 K. (**d**) M-H curves at 30 K.

Since a diamagnetic property is extremely small if applied magnetic field is high, superconductor becomes very vulnerable to external influences. The behavior does not change at 15 K and 20 K because H_*c*2_ of the temperatures are considerably high in MgB_2_. In addition, it may be considered that H_*c*2_ varies depending on the specimen in M-H curve method as shown in the Fig. 1 because a diamagnetic property varies greatly depending on the pinning state of defects in relatively high field (6.5 T). Nonetheless, it is certain that ultimate H_*c*2_ does not change. Generally, it was reported that H_*c*2_ of MgB_2_ is 20–30 T^7–9^. However, this is equivalent to the statement that H_*c*2_ of MgB_2_ is more than 20 T and the upper limit is unknown.

No matter how high H_*c*2_ was measured, it was the H_*c*2_ that was appropriate for the condition of measurement and the state of the specimen, thus it has its own meaning. However, it is also important to measure ultimate H_*c*2_ experimentally. In previous study, we have asserted that a ΔH = ΔB region is formulated if volume defects are many enough in volume defect-dominating superconductor, which is the region that increased applied magnetic field is the same as increasing magnetic induction^10^. Two conditions are suggested for that pinned fluxes have to be picked out from the volume defect, which are F_*pinning*_ <  F_*pickout*_ or the distance between pinned fluxes at a volume defect is equal to that of H_*c*2_. We have calculated H_*c*2_ using the theory with experimental results, and have obtained a fairly reasonable result. Thus, we would introduce the method of obtaining H_*c*2_ of superconductor based on a ΔH = ΔB region.

## Results

### A model for flux quanta distribution at H_*c*2_ and problems of measurement

**Figure 2:**
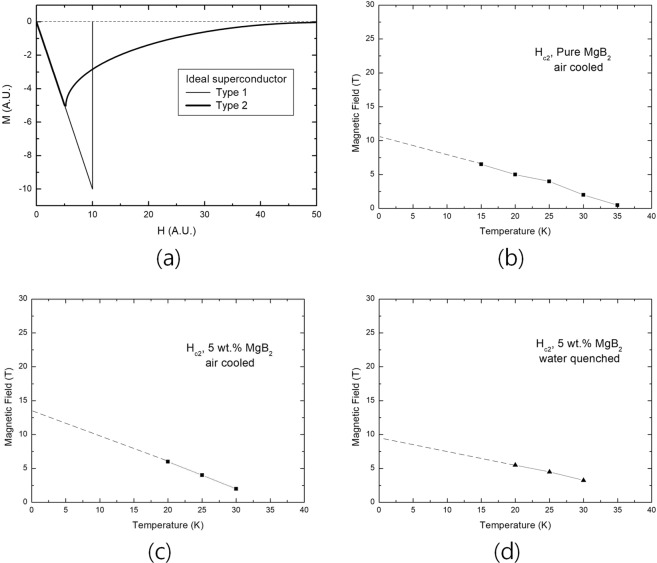
Field dependences of magnetization (M-H curve) for ideal superconductor and extrapolations of H_*c*2_ for various specimens. (**a**) H_*c*1_ and H_*c*2_ of ideal superconductor. (**b**) H_*c*2_ of pure MgB_2_, which was air-cooled. (**c**) H_*c*2_ of 5 wt.% (Fe, Ti) doped MgB_2_, which was air-cooled. (**d**) H_*c*2_ of 5 wt.% (Fe, Ti) doped MgB_2_, which was water-quenched.

 Figure 1 shows field dependences of magnetization (M-H curves) for various temperature, and applied magnetic field is the maximum at 6.5 T, and they are cut to focus H_*c*2_. M-H curves below 15 K are not considered because the diamagnetic properties at the temperatures are quite meaningful at 6.5 T. The field that diamagnetic property changes from  −  to + is H_*c*2_, and results are shown in Fig. 2. The equation of extrapolating is as follows.

1 $$\begin{array}{ccc}\xi {(T)}^{2}\propto \frac{1}{1-t}\Rightarrow {H}_{c2}=\frac{{\Phi }_{o}}{2\pi {\xi }^{2}}\propto 1-t &  & \end{array}$$

where t = *T*_*m*_/*T*_*c*_, *ξ* is coherence length^1,11^. *T*_*m*_ is the measuring temperature and *T*_*c*_ is critical temperature.

**Figure 3:**
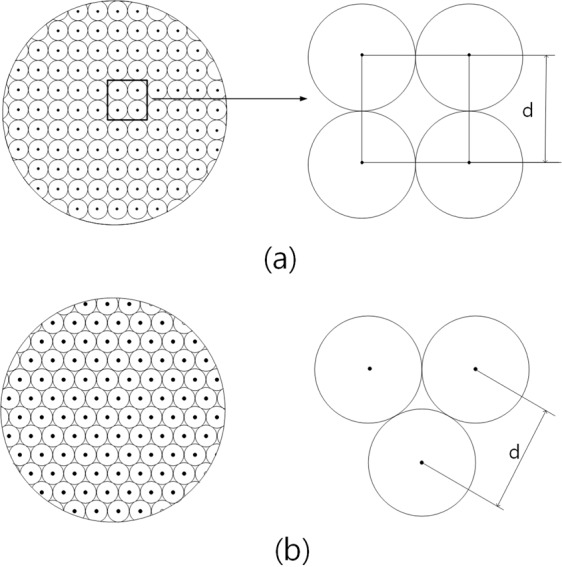
Schematic representations for an arrangement of flux quanta at H_*c*2_. (**a**) Square form of flux quanta at H_*c*2_. (**b**) Triangular form of flux quanta at H_*c*2_.

As shown in Fig. 2, different results were obtained for three specimens, and are needed to check that they were closer to the ultimate H_*c*2_ of the specimen. Since the diamagnetic properties of the superconductor approach zero if the external magnetic field is high enough, the arrangements of the flux quanta in the superconductor would be that of Fig. 3. When many magnetic fluxes quanta have penetrate into the inside of the superconductor, the supercurrent circulating the surface of the superconductor is the only one related to diamagnetic property. Thus, the field that superconducting current disappears would be H_*c*2_ as the external field increases.

As shown in Fig. 1, applied magnetic field increased by 0.5 T when the external field is above 1 T in doped specimens whereas it did by 0.25 T in pure specimen. It is clear that all of increased magnetic field penetrates the inside of the superconductor when the diamagnetic property is close to zero. On the other hand, the fluxes in the superconductor exist as flux quanta, and the repulsive forces are acting between them. The repulsive force per unit length (cm) between quantum fluxes is, which is caused by vortexes

2 $$\begin{array}{ccc}f={J}_{s}\times \frac{{\Phi }_{o}}{c} &  & \end{array}$$

where *J*_*s*_ is the total supercurrent density due to vortices^12^.

When a large field of 0.5 T increases at once, the flux quanta that try to penetrate into the superconductor will interact with the existing flux quanta in the superconductor, and the repulsive force between them will cause continuous vibration. The vibration will cause mutual interference, which are amplification and attenuation. The attenuation is expected to have little effect on the diamagnetic supercurrent, but the amplification may cause some flux quanta to rebound out from the superconductor because there is little difference in fluxes density between the inside and the outside of the superconductor and circulating supercurrent is tiny.

When rebounds of quantum fluxes occur, the supercurrents circulating the surface of the superconductor are interfered. When the number of the rebounding flux quantum exceeds a certain value, the supercurrents would disappear, which induces that the diamagnetic property does not appear. It is considered that the phenomenon increases as the distance between fluxes become closer to that of ultimate H_*c*2_, and increases as the magnitude of magnetic field applied at once increases. Therefore, it is clear that H_*c*2_ obtained from M-H curves must be much lower than the ultimate H_*c*2_.

On the other hand, when the magnetic field is applied first to the superconductor and next the superconducting current is supplied to determine H_*c*2_ of a superconductor, fluxes inside the superconductor would be much more stabilized. In this case, it is determined that the magnetic field in the superconductor is arranged in the form of Fig. 3(a) or ultimately stabilized form of Fig. 3(b). From the point of view, it is considered that the arrangement of the flux quanta in the H_*c*2_ state of a superconductor is not depending on what kind of superconductor is, but how long it takes after applying the magnetic field^13,14^. Since a stabilization of quantum fluxes would be achieved over time, it is natural that the arrangement of the flux quanta would be the state as shown in Fig. 3(b).

If a small amount of current flows around the surface of specimen in the state that the magnetic fluxes in the superconductor are stabilized, the increasing magnetic flux quanta in the superconductor is only a magnetic field generated by the flowing current. If the magnitude of the current is small, the interference caused by the increased magnetic flux quanta would be small, thus the magnetic flux quanta rebounding out of the superconductor will also be small. Therefore, it is considered that H_*c*2_ measured by currents method is much closer to the ultimate H_*c*2_ of the superconductor than that of M-H curves. However, it is certain that H_*c*2_ measured by this method is not the ultimate because a certain amount of current must flow, which generates a magnetic field.

### Upper critical field by ΔH = ΔB region

If volume defects are spherical, their size is constant, and they are arranged regularly in a superconductor, a superconductor of 1 cm^3^ has m′^3^ volume defects. Assuming that the pinned fluxes at volume defects are picked out and move into an inside of the superconductor when the distance between pinned fluxes is the same as that of H_*c*2_ as shown in Fig. 3(a), the maximum number of flux quanta that can be pinned at a spherical defect of radius *r* in a static state is

3 $$\begin{array}{ccc}{n}^{2}=\frac{\pi {r}^{2}}{\pi {\left(\frac{d}{2}\right)}^{2}}\times P={\left(\frac{2r}{d}\right)}^{2}\times P=\frac{\pi {r}^{2}}{{d}^{2}} &  & \end{array}$$

where *r*, *d* and *P* is the radius of defects, the distance between quantum fluxes pinned at the volume defect of which radius is *r* and filling rate which is *π*/4 when they have square structure, respectively, as shown in Fig. 3(a)^15^.

If volume defects in a superconductor are many enough, the superconductor has a ΔH = ΔB region, and the width of the region is

4 $$\begin{array}{ccc}{W}_{\Delta H=\Delta B}={H}_{final}-{H}_{c1}^{{\prime} }={n}^{2}{m}_{cps}m{\Phi }_{0}-4\pi M-{H}_{c1}^{{\prime} } &  & \end{array}$$

where *H*_*final*_ is the final field of the ΔH = ΔB region, ${H}_{c1}^{{\prime} }$ is the first field of the ΔH = ΔB region^10^. *m*_*cps*_ is the number of defects which are in the vertically closed packed state, *n*^2^ is the number of flux quanta pinned at a defect of radius r, *m* is the number of the volume defects from surface to center along an axis (*m*′ = 2*m*), *M* is magnetization, and Φ_0_ is flux quantum. *m*_*cps*_ is the minimum number of defects when the penetrated fluxes into the superconductor are completely pinned. Thus, 2*r* × *m*_*cps*_ is unit.

The number of flux quanta pinned at a defect is

5 $$\begin{array}{ccc}{n}^{2}=\frac{{W}_{\Delta H=\Delta B}+4\pi M+{H}_{c1}^{{\prime} }}{{m}_{cps}m{\Phi }_{0}} &  & \end{array}$$

Arranging after the equation is put into Eq. (3),

6 $$\begin{array}{ccc}{d}^{2}=\frac{\pi {r}^{2}{m}_{cps}m{\Phi }_{0}}{{W}_{\Delta H=\Delta B}+4\pi M+{H}_{c1}^{{\prime} }} &  & \end{array}$$

Therefore

7 $$\begin{array}{ccc}{H}_{c2}=\frac{{\Phi }_{0}}{{d}^{2}}=\frac{({W}_{\Delta H=\Delta B}+4\pi M+{H}_{c1}^{{\prime} })}{\pi {r}^{2}{m}_{cps}m} &  & \end{array}$$

Because 2*r* × *m*_*cps*_ = 1, the equation is

8 $$\begin{array}{ccc}{H}_{c2}=\frac{{\Phi }_{0}}{{d}^{2}}=\frac{2({W}_{\Delta H=\Delta B}+4\pi M+{H}_{c1}^{{\prime} })}{\pi rm} &  & \end{array}$$

**Figure 4:**
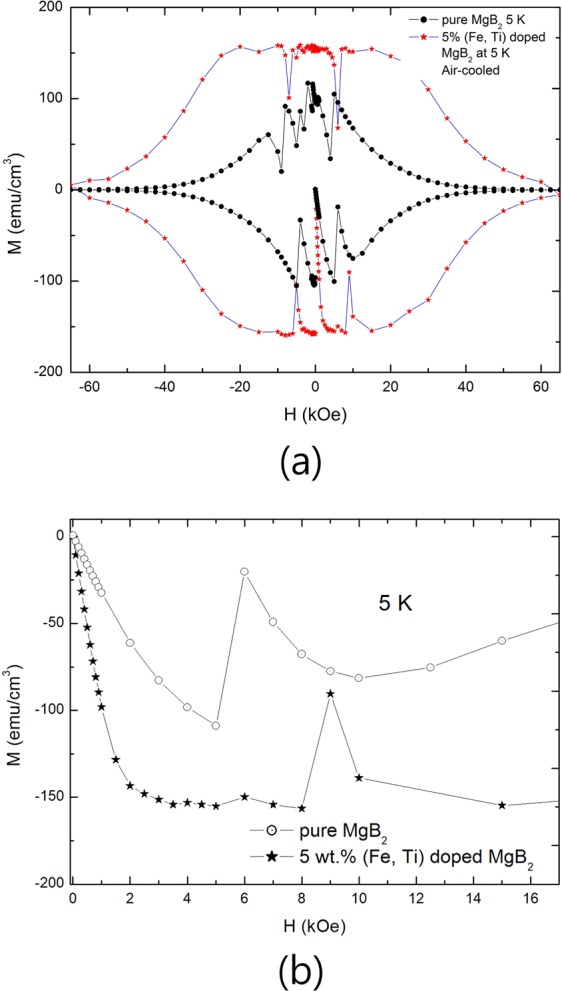
A decision of the width of the ΔH = ΔB region from field dependences of magnetization (M-H curves) for pure MgB_2_ and 5 wt.% (Fe, Ti) doped MgB_2_. (**a**) Full M-H curves. (**b**) Zero width of the ΔH = ΔB region in pure MgB_2_ and 1.3 T (1.5 T  −  0.2 T) of ΔH = ΔB region in 5 wt.% (Fe, Ti) doped MgB_2_, which was air-cooled.

The thickness of the specimen used for the measurement is 0.25 cm. Thus, the width of the region as unit length have to be 4 × t (t is the thickness of the specimen). In addition, since applied magnetic field penetrates into both sides of the specimen, volume defects inside the superconductor have pinned the fluxes for both side until applied magnetic field reach $H{}_{c1}^{{\rm{{\prime} }}}$. Therefore, the width of the region as unit length is

9 $$\begin{array}{ccc}{W}_{\Delta H=\Delta B}={n}_{d}w+({n}_{d}-1){H}_{c1}^{{\prime} } &  & \end{array}$$

where *w* is experimentally obtained width of the region, *n*_*d*_ is the number of specimen when a specimen of unit length was divided (*n*_*d*_*t* = 1). Although the width of the region is 1.3 T as shown in the Fig. 4(b), the width of ΔH = ΔB region as unit length is 5.8 T because the width of the specimen was 0.25 cm.

If the average radius of defects, the width of ΔH = ΔB region, *M*, $H{}_{c1}^{{\rm{{\prime} }}}$, and *m* are 163 nm, 5.8 T, −150 emu/cm^3^, 2000 Oe, and 4000, respectively, which are experimental results of 5 wt.% (Fe, Ti) particle-doped MgB_2_ as shown in Fig. 4(a,b), H_*c*2_ of the specimen is 56.7 T at 5 K. Concerning *m*, it is 4000 because magnetic field penetrates into the superconductor from both sides although the specimen have 8000^3^ volume defects of average 163 nm radius^10^. The coherence length (*ξ*) is 2.41 nm when H_*c*2_ is 56.7 T at 5 K. Extrapolated by Eq. (1), *ξ* is 2.24 nm and H_*c*2_ is 65.4 T at 0 K. Accidentally, the value is much closer to that of Ginzburg-Landau theory, which is 68.6 T at 0 K^6^.

### Discussion

As mentioned earlier, the methods of measuring H_*c*2_ of a superconductor have their own drawbacks. Supercurrents method may be close to the ultimate H_*c*2_ of the superconductor, but it is clear that there is a difference between the result and the ultimate H_*c*2_ because of the magnetic field induced by applied currents. However, we believe that H_*c*2_ measured by this method can further reduce the difference.

We could understand how stabilized the pinned fluxes are in H_*c*2_ state if inspecting the force balances of the pinned fluxes when they are picked out. Generally, pinned fluxes at volume defect move when F_*pickout*_ is more than ΔF_*pininng*_. However, it was our assertion that the pinned fluxes are picked out and moved even in F_*pinning*_ > F_*pickout*_ state when the distance between them is equal to that of H_*c*2_. The justification of the assumption is that there is no pinning effect if the neighborhoods of the volume defect are changed to normal state.

F_*pinning*_ is

10 $$\begin{array}{ccc}{F}_{pinning}=\frac{{\rm{\partial }}G}{{\rm{\partial }}r}=-\frac{{H}_{c1}^{{\prime} 2}}{8\pi }\times 4\pi {r}^{2}+\frac{2{n}^{2}{\Phi }_{o}^{2}}{8\pi } &  & \end{array}$$

and F_*pickout*_ is

11 $$\begin{array}{ccc}\Delta {F}_{pickout}=\frac{aL{H}_{c2}{\Phi }_{o}}{4\sqrt{P}cr}{n}^{4} &  & \end{array}$$

where *n*^2^ is the number of quantum fluxes pinned at a spherical volume defect of radius *r*, *H*_*c*2_ is upper critical field of the superconductor, Φ_*o*_ is flux quantum which is 2.07 × 10^−7^ G⋅cm^2^, *c* is the velocity of light, *aL* is an average length of quantum fluxes which are pinned and bent between defects (a is an average bent constant which is 1 < *a* < 1.2 and *L* is the distance between defects in vertically packed state) and P is the filling rate which is *π*∕4 when flux quanta are pinned at a volume defect in the form of square^15^.

Numerically, if $H{}_{c1}^{{\rm{{\prime} }}}$ is 2000 Oe, *r* is 0.163 nm, *n* is 45, and *aL* is 1.1 × 3.9 × 10^−4^ cm, which are results of idealized 5 wt.% (Fe, Ti) doped MgB_2_ specimen, F_*pinning*_ is 5.3 × 10^−4^ dyne and F_*pickout*_ is 1.4 × 10^−4^ dyne. Comparing F_*pinning*_ with F_*pickout*_, F_*pinning*_ /F_*pickout*_ is more than 4. Generally, when fluxes are approaching a volume defect, they have a velocity. If F_*pinning*_ are similar with F_*pickout*_, the pick-out of pinned fluxes from the volume defect is easier than that of calculation because fluxes have a velocity when they move in the superconductor. However, if F_*pinning*_ is more than 4 times of F_*pickout*_, it is considered that the depinning occurs after the distance between pinned fluxes is same as that of H_*c*2_ even if fluxes had some velocity.

## Conclusion

We have investigated characteristics of several methods for obtaining H_*c*2_ of type II superconductors and explained that any experimental method to obtain H_*c*2_ would be different from the ultimate H_*c*2_. In addition, no matter how high H_*c*2_ was obtained, it has its meaning because it was affected by the state of the specimen and measurement conditions. We suggested a method to obtain H_*c*2_, which is that H_*c*2_ of volume defect-dominating superconductor could be obtained from a width of ΔH = ΔB region. We used the property that ΔH = ΔB region is formed in the M-H curve when volume defects in the superconductor are many enough. It is based on the theory that pinned fluxes at the volume defects would be picked out from the volume defects and move when the distance between them is equal to that at H_*c*2_. From the results of 5 wt.% (Fe, Ti) doped MgB_2_, H_*c*2_ was 56.7 T at 5 K, which is quite same as that of Ginzburg-Landau theory. We obtained that F_*pinning*_/F_*pickout*_ is more than 4 in ΔH = ΔB region, which means that fluxes had been pinned at the volume defect were depinned even though F_*pinning*_ is much larger than F_*pickout*_. The behavior means that the H_*c*2_ is less sensitive to fluctuation. Therefore, it is determined that the obtained H_*c*2_ by the method is much closer to the ultimate H_*c*2_ of the superconductor.

## Method

Pure MgB_2_ and (Fe, Ti) particle-doped MgB_2_ specimens were synthesized using the nonspecial atmosphere synthesis (NAS) method^16^. Briefly, NAS method needs Mg (99.9% powder), B (96.6% amorphous powder), (Fe, Ti) particles and stainless steel tube. Mixed Mg and B stoichiometry, and (Fe, Ti) particles were added by weight. They were finely ground and pressed into 10 mm diameter pellets. (Fe, Ti) particles were ball-milled for several days, and average radius of (Fe, Ti) particles was approximately 0.163 *μ*m^10^. On the other hand, an 8 m-long stainless-steel (304) tube was cut into 10 cm pieces. Insert holed Fe plate into stainless- steel (304) tube. One side of the 10 cm-long tube was forged and welded. The pellets and pelletized excess Mg were placed at uplayer and downlayer in the stainless-steel tube, respectively. The pellets were annealed at 300 °C for 1 hour to make them hard before inserting them into the stainless-steel tube. The other side of the stainless-steel tube was also forged. High-purity Ar gas was put into the stainless-steel tube, and which was then welded. Specimens had been synthesized at 920 °C for 1 hour. They are cooled in air and quenched in water respectively. The field and temperature dependence of magnetization were measured using a MPMS-7 (Quantum Design).
